# Structural equation modeling of factors contributing to quality of life in Japanese patients with multiple sclerosis

**DOI:** 10.1186/1471-2377-13-10

**Published:** 2013-01-22

**Authors:** Hiromi Kikuchi, Nobuhiro Mifune, Masaaki Niino, Jun-ichi Kira, Tatsuo Kohriyama, Kohei Ota, Masami Tanaka, Hirofumi Ochi, Shunya Nakane, Seiji Kikuchi

**Affiliations:** 1 College of Nursing, Sapporo City University, Sapporo, Japan; 2 Japan Society for the Promotion of Science, Kobe University, Kobe, Japan; 3 Department of Clinical Research, Hokkaido Medical Center, Yamanote 5jo 7chome, Nishi-ku, Sapporo, 063-0005, Japan; 4 Department of Neurology, Neurological Institute, Graduate School of Medical Sciences, Kyushu University, Fukuoka, Japan; 5 Department of Neurology, Hiroshima City Hospital, Hiroshima, Japan; 6 Faculty of Science, Tokyo University of Science, Tokyo, Japan; 7 MS Center, Utano National Hospital, Kyoto, Japan; 8 Department of Geriatric Medicine & Neurology, Ehime University School of Medicine, Ehime, Japan; 9 Department of Clinical Research, Nagasaki Kawatana Medical Center, Kawatana, Japan; 10 Department of Neurology, Hokkaido Medical Center, Sapporo, Japan

**Keywords:** Multiple sclerosis, Quality of life, Structural equation modeling, Severity, Treatment, and Intervention

## Abstract

**Background:**

To improve quality of life (QOL) in patients with multiple sclerosis (MS), it is important to decrease disability and prevent relapse. The aim of this study was to examine the causal and mutual relationships contributing to QOL in Japanese patients with MS, develop path diagrams, and explore interventions with the potential to improve patient QOL.

**Methods:**

Data of 163 Japanese MS patients were obtained using the Functional Assessment of MS (FAMS) and Nottingham Adjustment Scale-Japanese version (NAS-J) tests, as well as four additional factors that affect QOL (employment status, change of income, availability of disease information, and communication with medical staff). Data were then used in structural equation modeling to develop path diagrams for factors contributing to QOL.

**Results:**

The Expanded Disability Status Scale (EDSS) score had a significant effect on the total FAMS score. Although EDSS negatively affected the FAMS symptom score, NAS-J subscale scores of anxiety/depression and acceptance were positively related to the FAMS symptom score. Changes in employment status after MS onset negatively affected all NAS-J scores. Knowledge of disease information improved the total NAS-J score, which in turn improved many FAMS subscale scores. Communication with doctors and nurses directly and positively affected some FAMS subscale scores.

**Conclusions:**

Disability and change in employment status decrease patient QOL. However, the present findings suggest that other factors, such as acquiring information on MS and communicating with medical staff, can compensate for the worsening of QOL.

## Background

Multiple sclerosis (MS) is a chronic disease of the central nervous system that manifests as inflammation, demyelination, axonal degeneration, and gliosis. To date, however, no known cure for the disease exists, and patients with MS may experience a number of neurological disabilities despite receiving adequate disease-modifying therapies. The disabling nature of the disease has multiple consequences which not only affect physical activity, psychological aspects, and social interplay, but also create burdens for family members [1]. Role limitations and cognitive and emotional problems have a negative impact on the health-related quality of life (QOL) of patients that is just as significant as the impact of physical symptoms or disability status [2]. A previous paper suggested that cognitive impairment and depression/anxiety decrease health-related QOL [3].

We previously reported the first evaluation of the entire QOL spectrum in Japanese MS patients by using the Functional Assessment of MS (FAMS), which consists of an MS-specific QOL scale with a self-reported questionnaire and the Nottingham Adjustment Scale-Japanese version (NAS-J), which is a self-reported questionnaire for determining psychological adaptation [4]. Out investigation revealed that FAMS subscale scores for mobility, symptoms, emotional well-being, thinking and fatigue, and additional concerns correlated strongly and negatively with the Expanded Disability Status Scale (EDSS) score, which is a measure of physical disorder in MS patients [4]. Moreover, additional factors affecting QOL (employment status, change of income, availability of disease information, and communication with medical staff)––as identified by MS patients in a focus group interview––were also investigated, and the first three factors were found to be important for maintaining the QOL of MS patients [4]. Overall, environmental and social factors, as well as the disabilities directly due to MS, were found to affect the QOL of Japanese MS patients [4]. Other studies have reported similar psychosocial and social effects on the QOL of MS patient. These factors include gender [5,6], employment status [7,8], income [9], education [10], and economic burden [11,12].

To improve QOL in MS, it is important to decrease disability and prevent relapse. Therefore, in this study we used structural equation modeling for the following purposes: 1) to examine the causal and mutual relationships that contribute to QOL in Japanese MS patients; and 2) to develop path diagrams for determining the interventions that are useful for targeting the environmental factors with the potential to improve QOL.

## Methods

### Sample and data collection procedures

The study population of the present study is the same as that of our previous study [4]. Briefly, subjects comprised 163 Japanese patients with MS (118 females, 45 males) at the following eight hospitals: Kyushu University, Tokushima University, Hiroshima University, Utano National Hospital, Nagoya University, Tokyo Women’s Medical University, Niigata University, and Hokkaido University. Mean age at onset of MS was 31.9 years, and mean duration of the disease was 10.4 years. Mean score on the Expanded Disability Status Scale (EDSS) was 3.99.

### Ethics statement

The study protocol was approved by the Ethics Committee of Hokkaido University, and written consent was obtained from all patients.

### Measures

The measures completed by the MS patients as a means to assess QOL are described in detail in our previous paper [4]. In this study, we used the data obtained from the FAMS, the NAS-J, and four additional factors of QOL which were cited as important by MS patients who had attended a separate focus group interview. The FAMS consists of 7 subscales (mobility, symptoms, emotional well-being, general contentment, thinking and fatigue, family/social well-being, and additional concerns) comprising 58 items. Each item is scored on a 5-point scale (ranging from “not at all” to “very much”, or equivalent degrees to produce a score between 0 and 4. The NAS-J consists of 6 subscales (anxiety/depression, self-esteem, attitude, locus of control, acceptance, and self-efficacy) comprising 27 items, with each item scored on a 4-point scale (ranging from “not at all” to “very much”, or equivalent degrees), producing a score between 1 and 4 for each question. The assessment battery included the following four additional factors affecting QOL [4]: employment status (1 item); change of income (1 item); availability of disease information (3 items: number of sources of disease information, self-evaluation of the availability of sources of disease information, and self-evaluation of knowledge of disease information); and communication with doctors and nurses (1 item). The questionnaire items and response choices are shown in Table 1. Disease severity was determined using Kurtzke’s EDSS [13].

**Table 1:** Questionnaire items for the 4 additional factors pointed out by MS patients in the focus group interview

**Factor**	**Questionnaire item**	**Choice**
Employment status	What was your employment status before and after the onset of MS?	Unemployed
		Part-time
		Full-time
Income	Did your income increase or decrease after the onset of MS?	Increase
		No change
		Decrease
Disease information	How many sources do you have for obtaining information on MS?	
	(Number of sources of disease information)	
	Do you think that you have enough opportunities to obtain information on MS?	0. No
	(Self-evaluation of sources of disease information)	1. Yes, a little
		2. Yes, some
		3. Yes, quite a bit
		4. Yes, a lot
	How much do you know about MS?	0. Nothing
	(Self-evaluation of knowledge of disease information)	1. A little
		2. Some
		3. Quite a bit
		4. A lot
Communication with medical staff	Are you satisfied with communication with your doctor/nurse?	0. No
		1. Yes, a little
		2. Yes, somewhat
		3. Yes, quite a bit
		4. Yes, very much

### Analysis

Using SPSS® Amos, structural equation modeling was performed to develop path diagrams of the factors that contribute to QOL. Models were constructed with the additional four factors as independent variables, FAMS subscales as dependent variables, and NAS-J subscales as mediator variables between the independent and dependent variables.

When analyzing the total FAMS score as well as each FAMS subscale, the mean score of all FAMS subscales was taken to be the dependent variable, and the mean score of all NAS-J subscales was taken to be the mediator variable. Finally, the mediating effects of the NAS-J subscales were investigated by examining latent variables to determine whether any of the relationships among the measures had been incorrectly assumed during the modeling process.

Maximum likelihood estimation was used to estimate model parameters. During this process, we used the following three goodness-of-fit indices: results of a Chi-squared test (χ^2^, *p*), comparative fit index (CFI), and root mean square error of approximation (RMSEA). When parameters with a high goodness-of-fit were determined such that several models had high fitness indices, the most parsimonious model was selected based on the Akaike’s Information Criterion.

## Results

### Causal and mutual relationships with FAMS total score (Figure 1)

Goodness-of-fit values were as follows: χ^2^ (5) = 2.656, *p* = 0.753, CFI = 1.000, RMSEA = 0.000. The EDSS score affected the total FAMS score directly, and the direct negative influence of the EDSS score on the total FAMS score had the largest path coefficient compared with other factors. Changes in household income positively and directly affected the total FAMS score. Increases in the number of sources of disease information and self-evaluation of knowledge of disease information positively affected the total NAS-J score, while changes in employment status negatively affected the total NAS-J score. The total NAS-J score positively influenced the total FAMS score, and its influence was 1.5-fold greater than that of EDSS.

**Figure 1:**
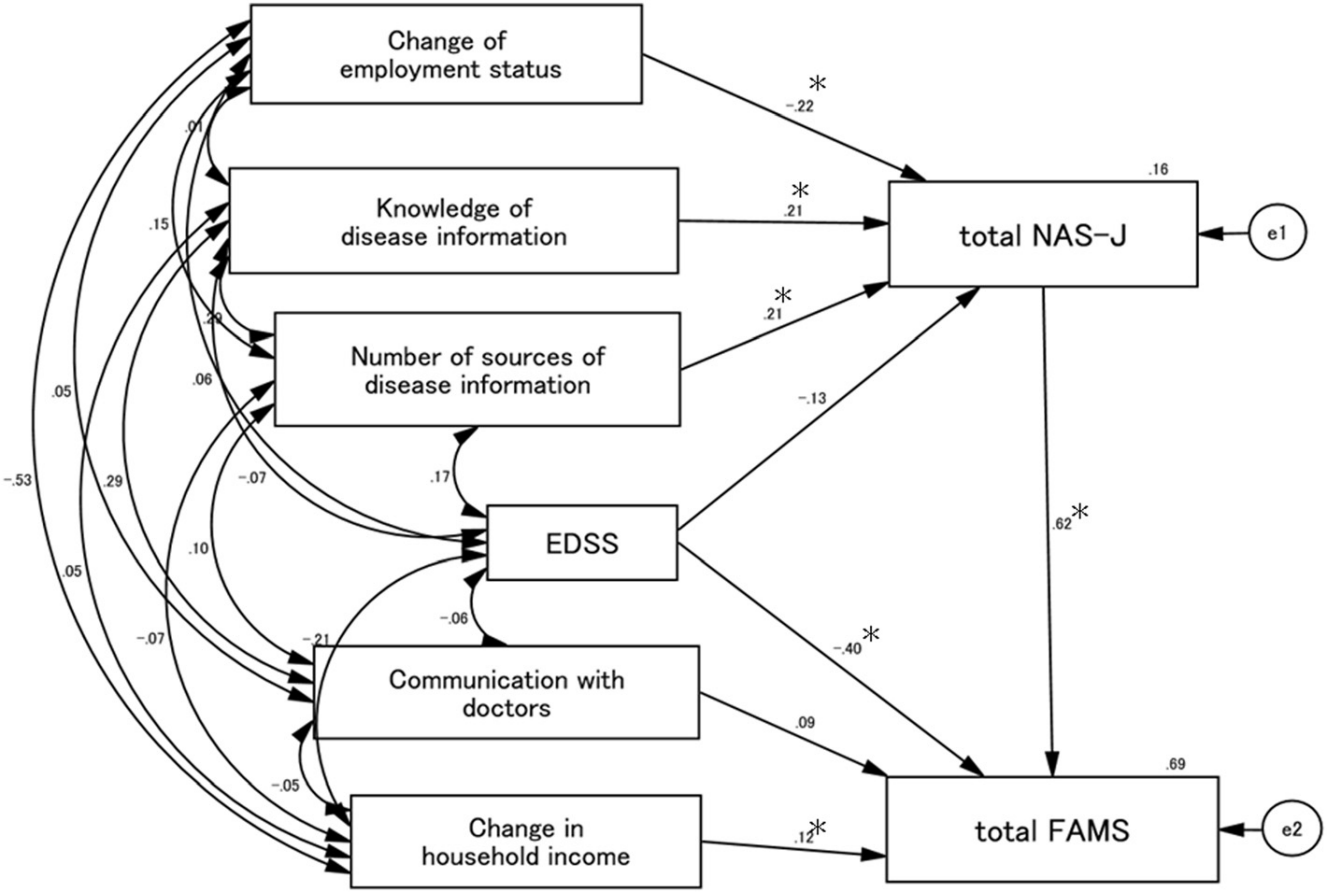
**Causal and mutual relationships with FAMS total score.** EDSS and changes in household income scores directly affect the total FAMS score. EDSS score negatively affect the total FAMS score, and the standardized total effect of EDSS on FAMS total score is -.48, which consists of a standardized direct effect of -.40 and a standardized indirect effect of -.08. On the other hand, changes in household income positively affect the total FAMS score. Changes in employment status negatively influence the total NAS-J score, while knowledge of disease information and number of sources of disease information have a positive influence. The latter factors indirectly affected the total FAMS score through the total NAS-J score. e1 and e2 in the figure are error terms. Numbers are standardized path coefficients, and asterisks (*) indicate statistical significance (*p* < 0.05).

### Causal and mutual relationships with FAMS mobility score (Figure 2)

Goodness-of-fit values were as follows: χ^2^ (7) = 7.129, *p* = 0.416, CFI = 1.000, RMSEA = 0.011. The EDSS score and changes in employment status decreased the scores of the latent variable comprising NAS-J anxiety/depression and acceptance. In contrast, self-evaluation of knowledge on MS positively affected these NAS-J subscales. A reduced EDSS score had a direct negative effect on the FAMS mobility score, and the path coefficient related to this influence was more negative than that for the effect of the EDSS score on the NAS-J anxiety/depression and acceptance scores. Finally, NAS-J anxiety/depression and acceptance scores positively affected FAMS mobility, although this positive effect was weak compared with the negative effect of increases on the EDSS score.

**Figure 2:**
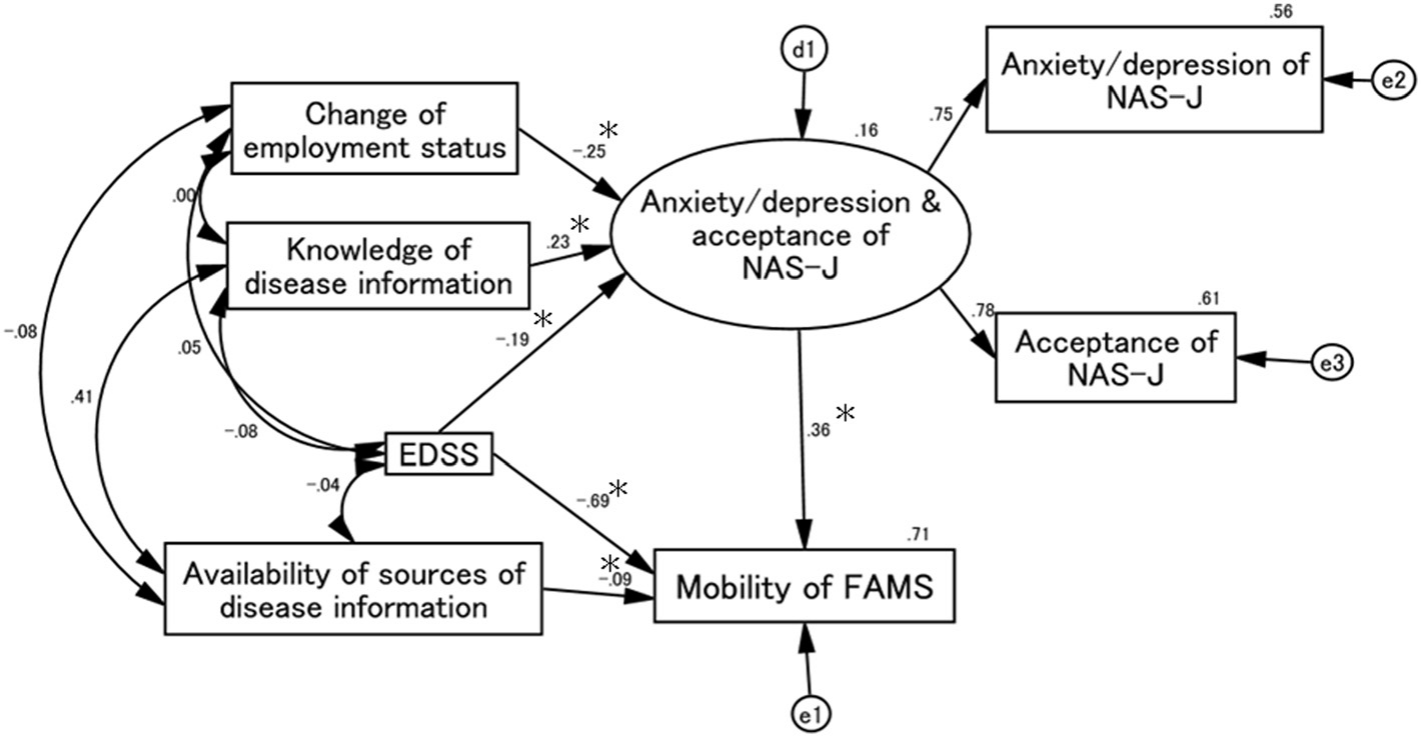
**Causal and mutual relationships with FAMS mobility score.** Increases in EDSS and availability of sources of disease information scores directly reduce the FAMS mobility score. The standardized total effect of EDSS on FAMS mobility score is -.76, which consists of a standardized direct effect of -.69 and a standardized indirect effect of -.07. Scores for changes in employment status and EDSS have a negative influence on the NAS-J anxiety/depression and acceptance scores. In contrast, knowledge of disease information scores positively affects the NAS-J anxiety/depression and acceptance scores. These three factors thus indirectly affect the FAMS mobility score through the NAS-J anxiety/depression and acceptance scores. d1, e1, e2, and e3 in the figure are error terms. Numbers are standardized path coefficients, and asterisks (*) indicate statistical significance (*p* < 0.05).

### Causal and mutual relationships with FAMS symptom score (Figure 3)

Goodness-of-fit values were as follows: χ^2^ (6) = 5.167, *p* = 0.523, CFI = 1.000, RMSEA = 0.000. Increased EDSS scores and changes in employment status reduced the scores of the latent variables comprising NAS-J anxiety/depression and acceptance, whereas the knowledge of disease information score increased these scores. However, knowledge of disease information negatively affected FAMS symptoms. Communication with doctors had a direct positive influence on the FAMS symptom score. The NAS-J anxiety/depression and acceptance scores had a positive effect on the FAMS symptom score, and its influence was 1.5-fold greater than that of EDSS. These data suggest that a decrease in patient anxiety/depression and acceptance of the disease (and its severity) can improve MS symptoms compared with an evaluation of patient disability using the EDSS.

**Figure 3:**
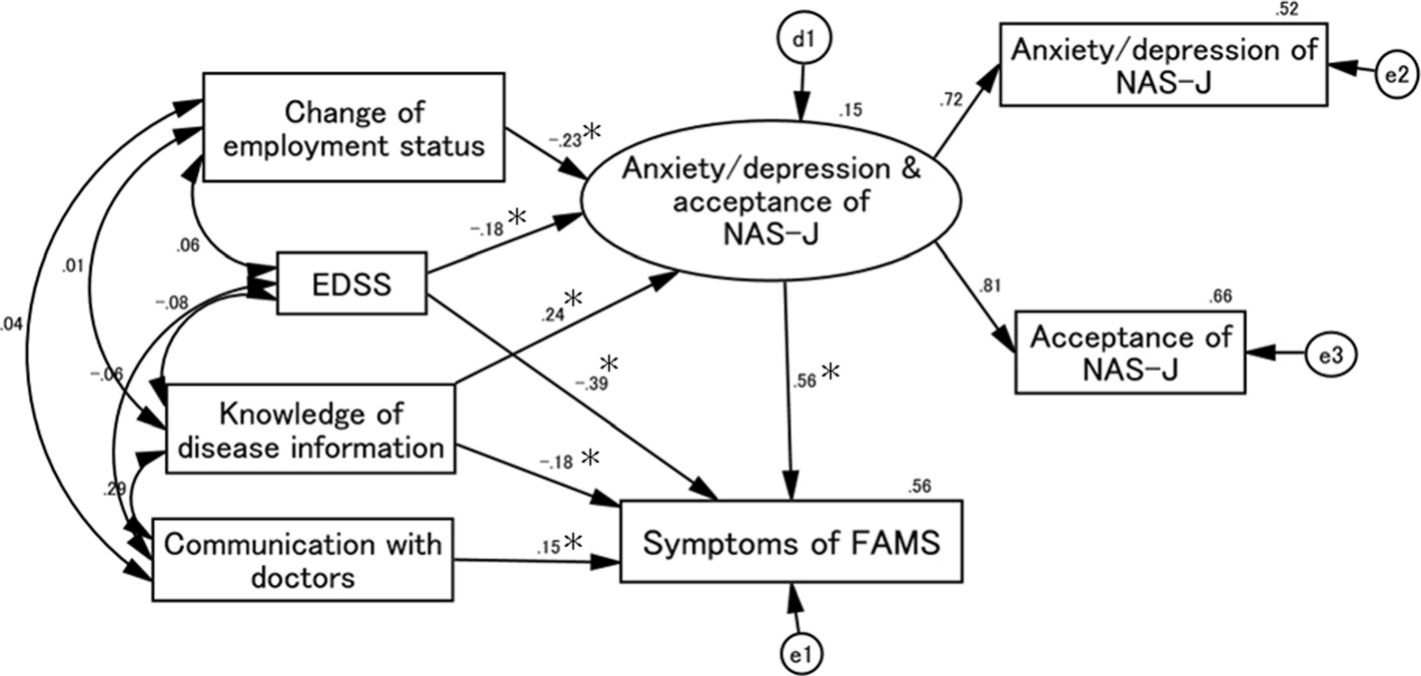
**Causal and mutual relationships with FAMS symptom score.** EDSS and knowledge of disease information scores have a direct negative affect on the FAMS symptom score. The standardized total effect of EDSS on FAMS symptom score is -.49, which consists of a standardized direct effect of -.39 and a standardized indirect effect of -.10. Conversely, communication with doctors has a direct positive influence on the FAMS symptom score. Increased scores for changes in employment status and EDSS reduce NAS-J anxiety/depression and acceptance scores, whereas knowledge of disease information scores positively affects these scores. Changes in employment status affect FAMS symptom score indirectly through the NAS-J anxiety/depression and acceptance scores. EDSS and knowledge of disease information scores affects FAMS symptom scores directly and indirectly through the NAS-J anxiety/depression and acceptance scores. d1, e1, e2, and e3 in the figure are error terms. Numbers are standardized path coefficients, and asterisks (*) indicate statistical significance (*p* < 0.05).

### Causal and mutual relationships with the FAMS emotional well-being score (Figure 4)

Goodness-of-fit values were as follows: χ^2^ (24) = 28.870, *p* = 0.225, CFI = 0.990, RMSEA = 0.035. Changes in employment status and EDSS scores negatively affected the NAS-J anxiety/depression, self-esteem, acceptance, and attitude scores. In contrast, these NAS-J subscales positively affected scores for the number of sources of disease information and self-evaluation of knowledge of disease information. Latent variables comprising the NAS-J anxiety/depression, self-esteem, acceptance, and attitude scores had a significant positive effect on the FAMS emotional well-being score. The personal income score also influenced the FAMS emotional well-being score, although the effect was weak.

**Figure 4:**
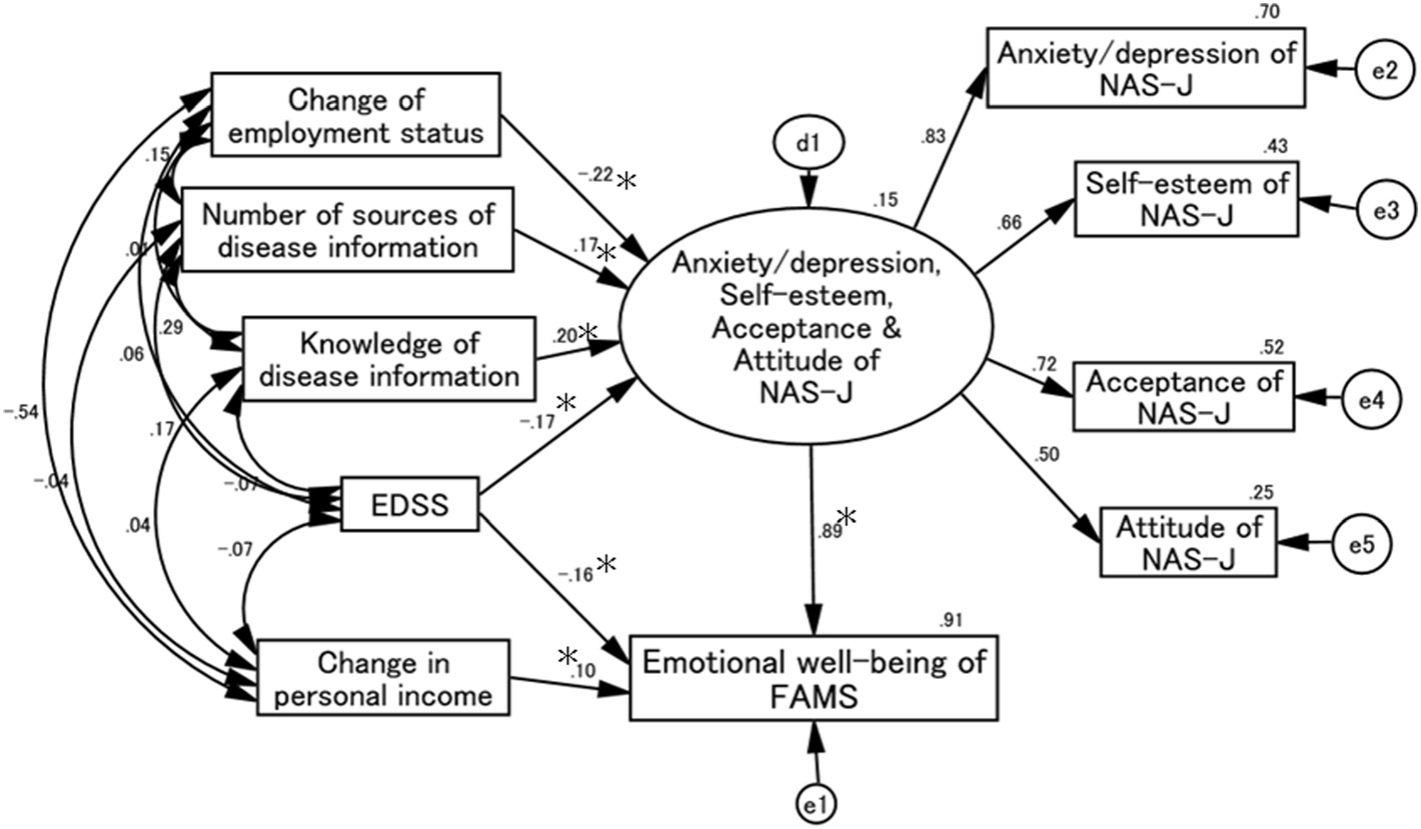
**Causal and mutual relationships with FAMS emotional well-being score.** The standardized total effect of EDSS on FAMS emotional well-being score is -.32, which consists of a standardized direct effect of -.16 and a standardized indirect effect of -.15. EDSS scores negatively affect the FAMS emotional well-being scores directly, while changes in personal income score positively affect this score directly. Changes in employment status and EDSS scores negatively influence NAS-J anxiety/depression, self-esteem, acceptance, and attitude scores as well as the number of sources of disease information score, and the knowledge of disease information score positively influences the NAS-J anxiety/depression, self-esteem, acceptance, and attitude scores. Changes in employment status, number of sources of disease information, and knowledge of disease information scores indirectly affect FAMS emotional well-being score through the NAS-J anxiety/depression, self-esteem, acceptance, and attitude scores. EDSS scores affect the FAMS emotional well-being score directly and indirectly through the NAS-J anxiety/depression, self-esteem, acceptance, and attitude scores. d1, e1, e2, e3, e4, and e5 in the figure are error terms. Numbers are standardized path coefficients, and asterisks (*) indicate statistical significance (*p* < 0.05).

### Causal and mutual relationships with FAMS general contentment score (Figure 5)

Goodness-of-fit values were as follows: χ^2^ (23) = 22.968, *p* = 0.463, CFI = 1.000, RMSEA = 0.000. All NAS-J subscale scores, except for attitude, positively affected the FAMS general contentment score. EDSS and changes in employment status scores negatively affected latent variables comprising NAS-J anxiety/depression, acceptance, and self-esteem scores, whereas increases in the self-evaluation of knowledge of disease information score positively affected these NAS-J subscale scores. Furthermore, increases in the score for the number of sources of disease information increased latent variables comprising NAS-J locus of control and self-efficacy scores. Here, the first set of the NAS-J latent variables comprising anxiety/depression, acceptance, and self-esteem scores produced a greater positive effect on the FAMS general contentment score compared with the second set which comprised the locus of control and self-efficacy scores.

**Figure 5:**
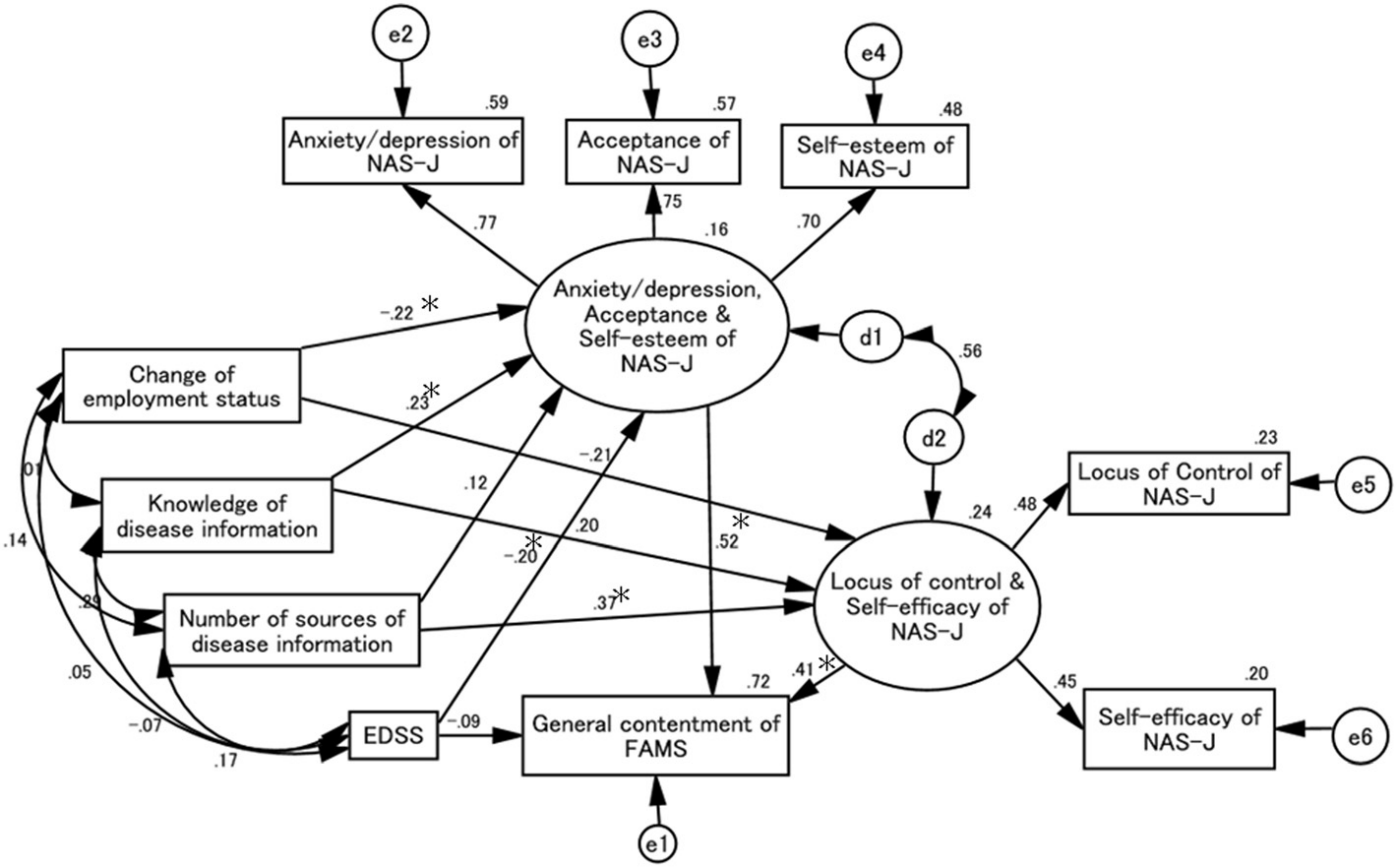
**Causal and mutual relationships with FAMS general contentment score.** The standardized total effect of EDSS on FAMS general contentment score is -.19, which consists of a standardized direct effect of -.09 and a standardized indirect effect of -.10. Changes in employment status and EDSS scores negatively affect the NAS-J anxiety/depression, acceptance, and self-esteem scores, whereas knowledge of disease information positively affects these subscales. Number of sources of disease information positively affects the NAS-J locus of control and self-efficacy scores. Changes in employment status, knowledge of disease information, number of sources of disease information, and EDSS scores affect the FAMS general contentment score indirectly through these NAS-J subscales. d1, d2, e1, e2, e3, e4, e5, and e6 in the figure are error terms. Numbers are standardized path coefficients, and asterisks (*) indicate statistical significance (*p* < 0.05).

### Causal and mutual relationships with FAMS thinking and fatigue score (Figure 6)

Goodness-of-fit values were as follows: χ^2^ (13) = 18.104, *p* = 0.154, CFI = 0.981, RMSEA = 0.049. NAS-J anxiety/depression, attitude, and acceptance scores positively affected the FAMS thinking and fatigue score. Conversely, increases in the EDSS and self-evaluation of knowledge of disease information scores decreased the FAMS thinking and fatigue score directly. Changes in household income and self-evaluation of knowledge of disease information scores related positively to the NAS-J anxiety/depression, attitude, and acceptance scores. However, the influence of the NAS-J anxiety/depression, attitude, and acceptance scores on the FAMS thinking and fatigue score was more than 2-fold that of EDSS.

**Figure 6:**
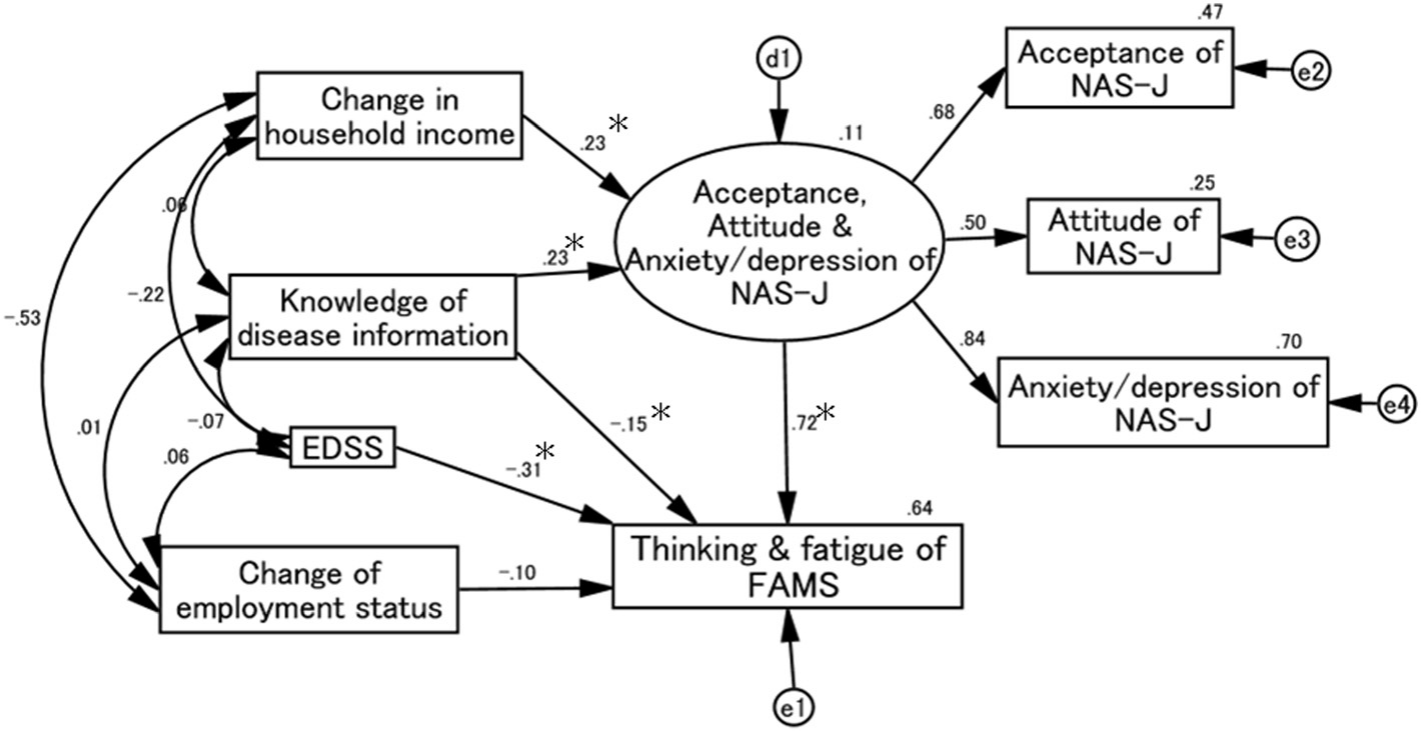
**Causal and mutual relationships with FAMS thinking and fatigue score.** Knowledge of disease information and EDSS scores negatively affect the FAMS thinking and fatigue score directly. Changes in household income and knowledge of disease information scores positively affect the NAS-J anxiety/depression, acceptance, and attitude scores and thus affect the FAMS thinking and fatigue score indirectly. d1, e1, e2, e3, and e4 in the figure are error terms. Numbers are standardized path coefficients, and asterisks (*) indicate statistical significance (*p* < 0.05).

### Causal and mutual relationships with FAMS family/social well-being score (Figure 7)

Goodness-of-fit values were as follows: χ^2^ (24) = 25.634, *p* = 0.372, CFI = 0.994, RMSEA = 0.021. Anxiety/depression, self-esteem, attitude, and acceptance scores all positively affected the FAMS family/social well-being score, and these NAS-J subscales were in turn negatively affected by changes in employment status and EDSS scores. Moreover, self-evaluation of knowledge of disease information scores positively influenced these NAS-J subscales. Scores for communication with nurses and the number of sources of disease information had a positive effect on the FAMS family/social well-being score. However, EDSS scores did not affect the family/social well-being score directly.

**Figure 7:**
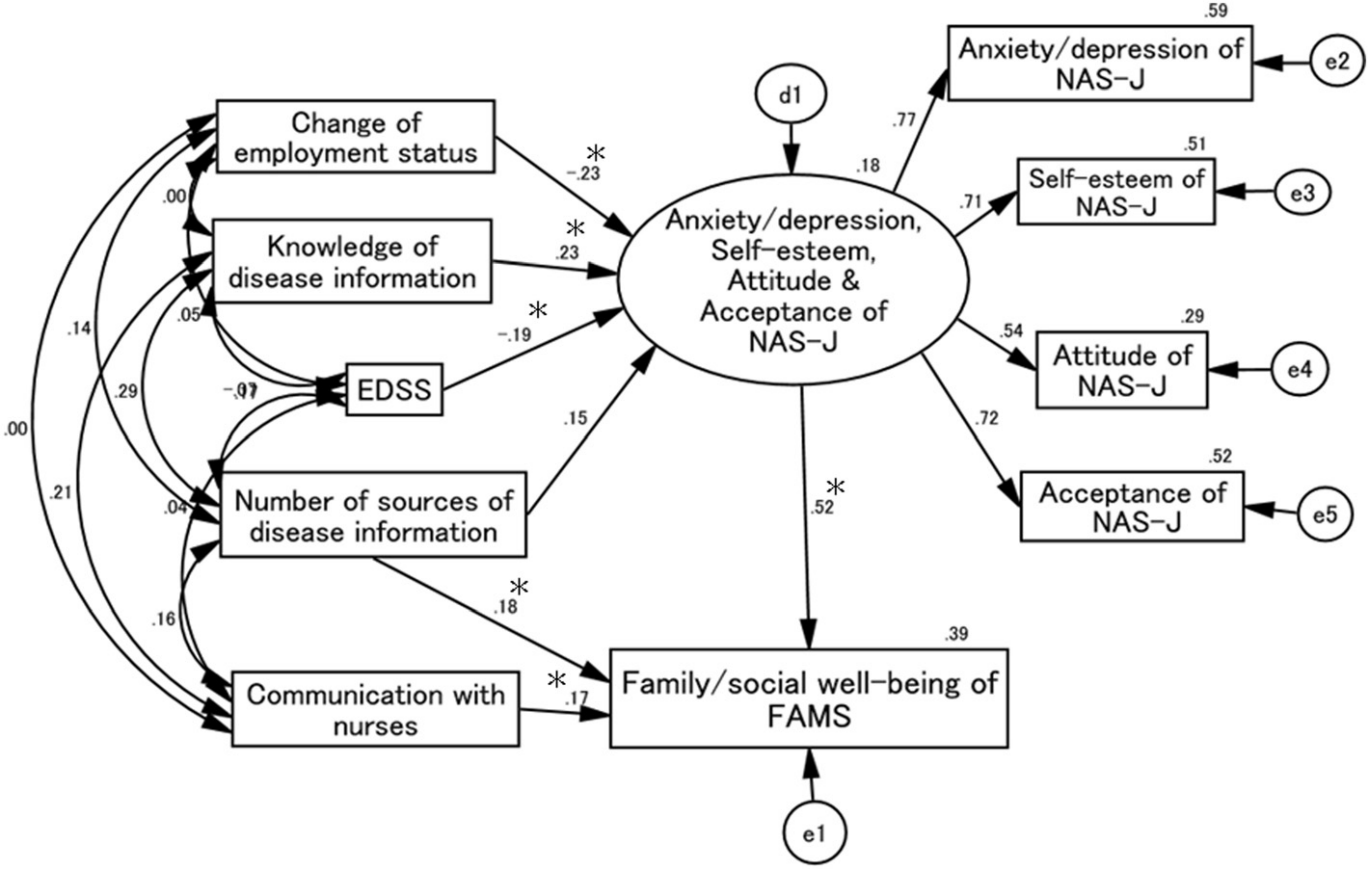
**Causal and mutual relationships with family/social well-being of FAMS score.** Scores for the number of sources of disease information and communication with nurses positively affect the FAMS family/social well-being score directly. Changes in employment status and EDSS scores negatively affect the NAS-J anxiety/depression, self-esteem, acceptance, and attitude scores. In contrast, knowledge of disease information positively influences these scores, which in turn have a positive influence on the FAMS family/social well-being score. d1, e1, e2, e3, e4, and e5 in the figure are error terms. Numbers are standardized path coefficients, and asterisks (*) indicate statistical significance (*p* < 0.05).

## Discussion

### EDSS and QOL

The EDSS score had the most prominent affect on the NAS-J subscale scores as well as on all the FAMS subscale scores both directly and indirectly. Worsening of disability negatively affected psychological status, which was followed by worsening of FAMS scores. Furthermore, the EDSS score directly affected mobility, symptoms, emotional well-being, and thinking and fatigue. Taken together, the EDSS score is an important reflection of the QOL of MS patients, which means that, for example, prevention of relapse and improvement of disability through rehabilitation could improve patient QOL [14,15]. Conversely, NAS-J subscales affected FAMS scores for symptoms, emotional well-being, general contentment, and thinking and fatigue to a greater extent than the EDSS score. It would appear then that improvement in these psychological factors can improve QOL, even if symptoms are deteriorating. Indeed, some studies have reported the success of cognitive behavior therapy for depression and fatigue in MS patients [16,17], and some psychological interventions might compensate for the worsening of QOL in MS patients.

### Changes in employment status and QOL

Changes in employment status after being diagnosed with MS reduced all NAS-J scores, and changes in employment status worsened patient anxiety, depression, acceptance of disability, and self-esteem scores. In contrast, obtaining more information on the disease compensated for worsening of the NAS-J scores in response to a change in employment status. We previously reported that 62% of patients experienced some kind of change in employment status, such as quitting a job or reducing their working hours, after the onset of MS [4]. The age at onset for most MS patients is in their 20s or 30s, and thus it is important for patients to continue working, which may require the support of others. Support to preserve income and obtain sufficient information on the disease can compensate for the decrement in QOL caused by a change in employment status.

### Information on MS and QOL

Obtaining information on MS improved the total NAS-J score, which was followed by improvement of many of the FAMS subscale scores. Increasing the number of sources of disease information also improved the scores for several NAS-J subscales. These findings indicate that acquiring information on MS is useful for psychological adjustment in MS patients and that awareness and coping may be associated with this improvement, as pointed out by Patti *et al.*[10]. In contrast, the increased availability of sources and knowledge on disease information worsened the FAMS mobility, symptoms, and thinking and fatigue scores directly, not through the total NAS-J score. The reason for this discrepancy between the NAS-J and FAMS scores is not easy to discern, but one possibility is that the important thing for MS patients is the quality––not quantity––of information on the disease. Patients may consider the disease or their disease status to be more serious than it is when they have inadequate information on the disease. It is therefore vital that MS patients are provided with adequate and appropriate information on the disease.

### Communication with medical staff and QOL

The effect of communication with medical staff differs between communication with doctors and that with nurses. Good communication with doctors improved the total FAMS score and FAMS symptom score directly, not through the total NAS-J score. Good communication with nurses also improved the FAMS family/social well-being score. The data suggest that good communication with medical staff can improve the QOL of MS patients while compensating for worsening disability. Moreover, it may be more effective for doctors to talk about symptoms and therapies with patients and for nurses to talk about daily life activities and self-care.

## Conclusions

The findings of the present study, which are in agreement with those of previous studies, indicate that the progression of disability and changes in employment status are major factors that decrease QOL for MS patients. While progression of disability may not be avoidable even in patients receiving adequate treatment, our findings suggest that other factors such as acquiring adequate information on MS and communication with medical staff can compensate for the decrease in QOL. Interventions that target these factors have the potential to improve QOL in Japanese MS patients.

## Abbreviations

MS: Multiple sclerosis; QOL: Quality of life; FAMS: The functional assessment of multiple sclerosis; NAS-J: The Nottingham adjustment scale-Japanese version; EDSS: The expanded disability status scale; CFI: The comparative fit index; RMSEA: The root mean square error of approximation.

## Competing interests

The authors declare that they have no competing interests.

## Authors’ contributions

HK designed the study and wrote manuscript. NM performed statistical analysis. MN assisted with writing the manuscript and reviewed the manuscript. JK, TK, KO, MT, HO, and SN contributed to data collection. SK assisted in study design and reviewed the manuscript. All authors read and approved the final manuscript.

## Pre-publication history

The pre-publication history for this paper can be accessed here:

http://www.biomedcentral.com/1471-2377/13/10/prepub
